# Augmented Renal Clearance and How to Augment Antibiotic Dosing

**DOI:** 10.3390/antibiotics9070393

**Published:** 2020-07-09

**Authors:** Iris H. Chen, David P. Nicolau

**Affiliations:** Center for Anti-Infective Research and Development, Hartford Hospital, 80 Seymour Street, Hartford, CT 06102, USA; ichen362@gmail.com

**Keywords:** augmented renal clearance, antibiotic, critical care, subtherapeutic, pharmacokinetics, pharmacodynamics

## Abstract

Augmented renal clearance (ARC) refers to the state of heightened renal filtration commonly observed in the critically ill. Its prevalence in this patient population is a consequence of the body’s natural response to serious disease, as well as the administration of fluids and pharmacologic therapies necessary to maintain sufficient blood pressure. ARC is objectively defined as a creatinine clearance of more than 130 mL/min/1.73 m^2^ and is thus a crucial condition to consider when administering antibiotics, many of which are cleared renally. Using conventional dosing regimens risks the possibility of subtherapeutic concentrations or clinical failure. Over the past decade, research has been conducted in patients with ARC who received a number of antibacterials frequently used in the critically ill, such as piperacillin-tazobactam or vancomycin. Strategies to contend with this condition have also been explored, though further investigations remain necessary.

## 1. Introduction

Augmented renal clearance (ARC) is the pathologic phenomenon wherein the kidneys display increased filtering activity beyond that expected under normal physiological conditions of renal function. Patients in this state have a creatinine clearance of more than 130 mL/min/1.73 m^2^ [1]. Although there are numerous pharmacologic agents that are potentially affected by ARC, this article will specifically explore its impact on antibiotics. This narrative review is intended to provide a contemporary assessment of antibiotic use in ARC patients from a pharmacokinetic/pharmacodynamic (PK/PD) perspective, including recently approved agents. Both the previous reviews and studies included herein demonstrate the long-standing challenges that originate from ARC, as well as the variety of antibiotics it affects. Clinical studies including patients with ARC remain uncommon. Thus, studies not including ARC patients have also been included when a demonstration of PK/PD principles is necessary. Most importantly, the strategies discussed to optimize dosing and exposure boil down to capitalizing on each drug’s driving PK/PD parameter while keeping therapeutic drug monitoring and alternative agents in mind, if needed.

In the clinical setting, the chief concern in treating a patient with ARC is the increased risk of subtherapeutic dosing and the subsequent clinical ramifications. While adjusting dosing regimens due to acute kidney injury or renal replacement therapies is second nature in clinical practice, the dose modifications for ARC are not so clear cut. More challenging is the lack of immediate response from underdosed antibiotics. Whereas the effects of drugs like vasopressors can be swiftly assessed and titrated to effect, the impact of insufficient antibacterials could very well manifest as microbiological or clinical failure, development of drug resistance, or death. Hence, it is vital to remain cognizant of ARC and the strategies available for treating patients with this condition.

## 2. Creatinine Clearance

Creatinine clearance serves as the objective parameter for determining whether a patient is experiencing ARC. Notably, however, multiple methods can be used to establish a value. Since urine collection studies are not generally a routine assessment, it is frequently estimated by using serum creatinine as a surrogate marker for renal function in conjunction with the Cockcroft–Gault (CG), Modification of Diet in Renal Disease (MDRD), or Chronic Kidney Disease Epidemiology Collaboration (CKD-EPI) equations (Table 1).

These equations are useful for providing renal function estimations quickly in patient care settings where time is of the essence. However, studies comparing the various formulas and their accuracies in different scenarios have been conducted, and varying conclusions prevent the establishment of a single, universally applicable calculation method [5,6,7,8,9]. The largest study, which included 24,516 adults with diabetes and compared calculated creatinine clearance with 24-h urine collections, concluded that the MDRD equation was overall the most accurate and least biased [9]. It also reported that the CG equation when calculated with ideal body weight, the CKD-EPI equation, and the MDRD equation underestimated the creatinine clearance, while the standard CG equation overestimated it. Despite these discrepancies, urine collection study results are not always available, and the required turnaround time should not delay dosing decisions or the administration of treatment. Hence, these equations remain useful in clinical practice.

Urine collection studies directly assess renal function by measuring creatinine content over specified time periods. While 24 h studies would supply the most complete picture of a patient’s status, they are not always practical, especially in a critical care setting where patients may need to be taken in for procedures or receive treatments capable of affecting filtration. Previously conducted studies suggest a minimum duration of 8 h [10,11,12]. In particular, Cherry and colleagues investigated the accuracy of collections performed over shorter durations in 100 critically ill trauma and surgical patients [10]. Urine collections were conducted over 2 h (CrCl_2_), 6 h (CrCl_6_), and 16 h (CrCl_16_). Creatinine clearance over 8 h (CrCl_8_) was determined by adding CrCl_2_ and CrCl_6_. Thereafter, CrCl_8_ was added to CrCl_16_ to obtain the 24 h CrCl (CrCl_24meas_), and a 24 h estimated CrCl (CrCl_24calc_) was calculated using the CG equation. The investigators ultimately recommended that urine studies be conducted over at least 8 h. Thus, 8 h urine collection studies are adequate for assessing renal function and should be carried out in catheterized patients, while calculating creatinine clearances should be reserved for situations wherein dosing decisions must be made without delay. In clinical practice, urine collection studies could be carried out overnight so that their results arrive with other daily morning labs and in time for dosing decisions to be made. Furthermore, they should be conducted regularly to assess whether the patient continues to have ARC and to readjust antibiotic dosing regimens accordingly.

## 3. Identifying Augmented Renal Clearance

**Figure 1:**
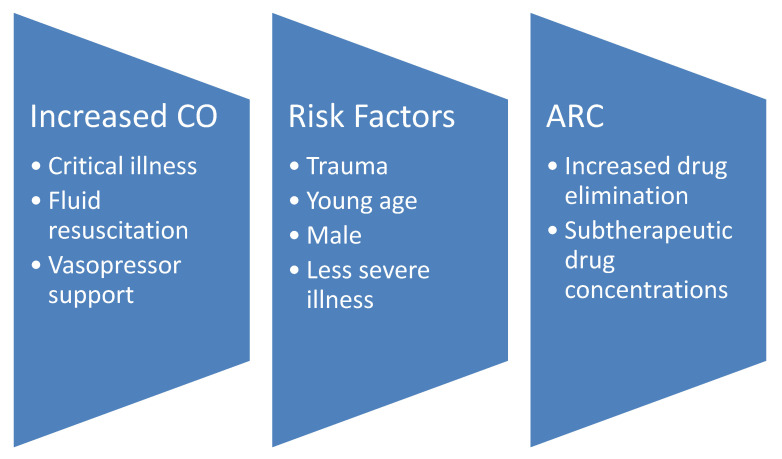
The development of augmented renal clearance (ARC); cardiac output, CO.

The definitive causes of ARC continue to remain unclear. As depicted in Figure 1, the chief theory is that it is a consequence of the body’s reaction to critical illness, fluid resuscitation therapy, and vasopressor support increasing cardiac output and, therefore, circulation through the kidneys [13,14]. Thus, ARC is frequently observed in critically ill patients. One systematic review observed that 20 to 65% of this population experiences ARC [1]. Commonly identified risk factors include trauma, young age, male sex, and less severe illness [1,13,15,16,17]. As discussed below, they form the foundations of the two scoring systems developed to evaluate the likelihood that a patient has ARC [13,18]. Other properties of note that have been associated with this condition are higher initial renal function, not having diabetes, and less frequent requirements for vasopressors [19,20]. Considered altogether, these factors should not come as a surprise. In a manner of speaking, these patients are the healthiest of the critically ill. For instance, any pre-existing conditions that affect renal function, such as chronic kidney disease or diabetes, would sooner put them at risk for decreased clearance and overdose as opposed to the other way around. That trauma is a major risk factor identified across multiple studies and the likely reason for intensive care unit (ICU) admittance to begin with could suggest that, by nature, the patient was an otherwise healthy person. ARC also occurs in pediatric patients. Van Der Heggen and colleagues observed that approximately two-thirds of the 105 critically ill pediatric study patients had ARC [21]. Similar to in the adult population, calculated estimates of renal function pale in comparison to direct measurement [21,22] and subtherapeutic concentrations are a natural consequence [22]. As will be discussed, the same strategies used to address ARC in adults can theoretically be used for pediatric patients.

Two major scoring systems have been developed to assess the likelihood of ARC in a patient. In 2013, a weighted ARC scoring system, hereafter referred to as ARC score, was created based on the findings from a prospective observational study involving 71 patients in the ICU [13]. Adult patients (18 to 80 years old) with a plasma creatinine greater than 110 µmol/L (approximately 1.24 mg/dL) were included if they were receiving either piperacillin-tazobactam for a nosocomial infection while meeting the Systemic Inflammatory Response Syndrome (SIRS) criteria or post-multi-trauma cefazolin prophylaxis. Urine collection was conducted to determine creatinine clearance and assess renal function. Cardiac index was also another primary parameter of interest. ARC was observed in 57.7% of the patients and major risk factors included younger age (≤50 years old), trauma, and a low modified sequential organ failure assessment (SOFA) score (≤4). These three factors were used to develop the ARC scoring system: patients age 50 and younger earn six points, trauma patients earn three points, and those with a modified SOFA score of no more than four earn one point. When grouping scores together, higher scores were found to signify a higher likelihood of ARC. This system also better predicted the presence of ARC than cardiac index on its own. Additionally, in a separate study, Akers and colleagues observed 100% sensitivity and 71.4% specificity for this scoring method [23].

A newer scoring system, the ARC in trauma intensive care (ARCTIC) score, was developed and published in 2017 based on the observations from a retrospective cohort study that included 133 patients admitted to the trauma ICU [18]. Similar to the ARC score study, patients were required to have undergone a timed urine collection assessment to accurately measure creatinine clearance. Additionally, their baseline serum creatinine could not be greater than 1.3 mg/dL and they were not allowed to have received renal replacement therapy. Barletta and colleagues conducted a multivariate analysis and found that younger age, low serum creatinine (<0.7 mg/dL), and male sex were major risk factors. The following rules comprise the ARCTIC scoring system: patients age 56 and younger receive four points, those ages 56 to 75 receive three points, those with a serum creatinine of less than 0.7 mg/dL receive three points, and male patients are assigned two additional points. Based on the sensitivity (0.843) and specificity (0.682) within the model, a score of six or higher was chosen as the ARCTIC score cutoff for consideration of adjustments to an antibiotic regimen. An advantage that the ARCTIC score has over the ARC score is the simplicity of calculation that does not require the inclusion of a SOFA score. On the other hand, it was specifically developed based on trauma ICU patients and has not yet been validated in a separate study. 

## 4. Effects on Antibiotic Therapy

One of the major defining features of every antibiotic is its driving PK/PD parameter. In other words, these are the specific concentration conditions that must be met for the agent to properly exert its effect on an infectious organism. Antibacterial activity relies on either time or concentration [24]. The minimum inhibitory concentration (MIC) of the causative bacteria is also vital for assessing antibiotic impact. Agents that display time-dependent activity do so as a function of time spent at a concentration above the MIC (% fT > MIC). Concentration-dependent antibiotic goals are expressed either as the ratio between the maximum achieved concentration and the MIC (C_max_/MIC) or the ratio between the area under the concentration curve and the MIC (AUC/MIC) [24]. Achieving these targets is made more difficult by the uncertainties posed by a patient with ARC and the lack of dosing guidance available for this patient population, which can translate into clinical consequences. In a study of 128 surgical and medical ICU patients receiving antimicrobial therapy, approximately half of which exhibited ARC, Claus and colleagues observed more therapeutic failure in the ARC group (27.3% vs. 12.9%, *p* = 0.04) [25]. Furthermore, subtherapeutic dosing may contribute to the development of antibiotic resistance [26].

The remainder of this section discusses observations in the treatment of ARC patients with beta-lactams, vancomycin, and other investigated compounds, as well as strategies for addressing this. Recommendations have been summarized in Table 2.

### 4.1. Beta-Lactams

Beta-lactams are time-dependent antibiotics and the minimum necessary % fT > MIC differs by class. Penicillin requires a % fT > MIC of 50% to 60% and cephalosporins need a % fT > MIC of 60% to 70%, while carbapenems have a slightly lower % fT > MIC threshold of 40% to 50% [27]. Despite the lack of routine testing for beta-lactam concentrations, the effects of enhanced kidney function during their use have been documented numerous times over the past decade [28,29,30,31,32]. In 2012, Udy and colleagues reported how elevated creatinine clearances of 130 mL/min/1.73 m^2^ resulted in a higher probability of subtherapeutic beta-lactam concentrations [28]. Another study previously observed suboptimal microbiological and clinical cure rates in bacteremia and septic patients receiving cefepime or ceftazidime when the 24-h area under the inhibitory curve was less than 250 or the % fT > MIC was less than 100% [29]. In a study involving critically ill patients with presumed sepsis, ARC was again observed to strongly predict undetectable imipenem, meropenem, piperacillin/tazobactam, and cefepime trough concentrations [30]. Given the common use of beta-lactams in the ICU [33,34,35], where potentially more than half the patients have ARC, regimens should be adjusted accordingly, and the maximum approved doses of the affected agents should be advocated as the standard of practice.

In the event that a patient must be maintained on an antibiotic impacted by ARC, it is vital to optimize the dosing regimen. Since enhanced clearance causes the subsequent subtherapeutic concentrations that endanger the patient, there are two major methods to addressing this matter. The first is to maximize the dosing regimen as much as possible. This is best done with agents with a wealth of historical data so that the drug can be administered safely. A cohort study of ARC patients in surgical and trauma ICUs was conducted to investigate whether receiving higher than standard beta-lactam dosing regimens was beneficial for an initial case of hospital- or ventilator-acquired pneumonia (HAP-VAP) [36]. In this study, ARC was defined as a creatinine clearance greater than 150 mL/min. A control period in which conventional intravenous regimens were used included the following: amoxicillin-clavulanic acid 2 g every 8 h, cefotaxime 2 g every 8 h, and ceftriaxone 2 g once daily. The treatment period then increased these regimens to amoxicillin-clavulanic acid 2 g every 6 h, cefotaxime 2 g every 6 h, and ceftriaxone 2 g every 12 h. The available broad-spectrum regimens remained the same between the two periods. They were comprised of piperacillin-tazobactam 16 g/day continuously, cefepime 6 g/day continuously, ceftazidime 6 g/day continuously, and meropenem 6 g/day continuously or 2 g every 8 h. The treatment period saw significantly lower therapeutic failure or HAP-VAP relapse (10 vs. 23%, *p* = 0.019) and no side antibiotic-related side effects were reported despite the increased regimens. It is reassuring to see how this study supports the use of such high doses in ARC patients. 

Another strategy is to adjust the administration time into prolonged or continuous infusions to take advantage of the nature of time-dependent antibiotics and % fT > MIC. In a 5000 patient Monte Carlo simulation, Kim and colleagues observed that patients with late-onset hospital-acquired pneumonia that required prolonged mechanical ventilation would receive optimal exposures of cefepime, ceftazidime, and meropenem when administered as prolonged infusions [37]. Another simulation study also demonstrated improved pharmacodynamics for prolonged and continuous infusions of piperacillin-tazobactam [38]. Since then, further studies have been carried out and expanded to include other beta-lactams. A randomized controlled trial in septic ICU patients not receiving renal replacement therapy concluded that continuous infusion of beta-lactams had better clinical cure rates and more frequently achieved PK/PD goals than intermittent dosing [39]. A pediatric study also concluded that extended and continuous infusion piperacillin dosing regimens allowed PK target attainment in ARC patients, as well as those with normal renal clearance, while standard intermittent regimens did not [40]. However, on its own, this strategy may not be sufficient. A substudy investigating the clinical outcomes of ARC patients in the Beta-lactam Infusion Group II (BLING-II) trial observed no statistically significant differences between those that received continuous beta-lactam infusions compared with those that received intermittent infusions [41]. Thus, it is important to combine it with other efforts, such as maximizing the dosage and utilizing therapeutic drug monitoring services when available. 

### 4.2. Vancomycin

Vancomycin is a well-known agent requiring therapeutic drug monitoring to both maximize clinical success and to minimize the risk of nephrotoxicity. The initial consensus guideline for this drug was made available in 2009 [42]. Traditionally, taking into consideration the complexity of calculating AUC/MIC and coordinating sample collection, trough concentrations of 15 to 20 mg/L were considered an adequate surrogate for the therapeutic AUC/MIC of 400 against methicillin-resistant *Staphylococcus aureus* (MRSA) infections with a maximum MIC of 1 mg/L and were sufficient for making dosing regimen adjustments. Additionally, a minimum trough concentration of 10 mg/L was encouraged to deter the development of resistance. Ideally, troughs were obtained before an upcoming dose once the patient had reached steady-state, or before the fourth dose. These goals were achieved through weight-based dosing and utilized actual body weight. The guideline suggested doses of 15 to 20 mg/kg administered every 8–12 h, with loading doses of 25 to 30 mg/kg being an option for critically ill patients requiring expedited attainment of therapeutic serum trough concentrations. 

Given its elimination by the kidneys and requisite monitoring, the effects of ARC on this drug have been thoroughly documented over the past decade. Studies agree that ARC enhances removal of vancomycin and reduces the likelihood of achieving a therapeutic trough, potentially endangering infected patients [43,44,45,46,47]. Villanueva and colleagues observed that only 16% of their 70 critically ill trauma patients initially achieved a therapeutic trough (14.5 to 20.5 mg/L) [45]. Upon performing a subgroup analysis for those with initial troughs of less than 10 mg/L (43% of the original group), they found that these patients also had significantly higher ARCTIC scores, lower initial median serum creatinine, and higher initial median creatinine clearance (as calculated by the CG equation). Another study compared the proportion of patients with subtherapeutic trough concentrations (<10 mg/L) between two groups, patients with and without ARC, and observed that they were significantly different (68.8% vs. 32.8%, *p* < 0.0001) [46]. Similar findings for vancomycin troughs of less than 10 mg/L were observed between the ARC and non-ARC groups in a pediatric study (79% vs. 52%, *p* < 0.001) [48]. Insufficient dosing negatively impacts clinical outcomes. In a study involving bacteremia caused by MRSA, patients that did not reach therapeutic AUC/MIC thresholds within the first two days of therapy were at twice the risk of failure (30-day mortality, bacteremia for seven or more days, or recurrence) [49]. 

Dosing and monitoring recommendations for vancomycin have recently undergone major revisions and appear to consider the possibility of patients with ARC. According to the 2020 guideline published by the American Society of Health-System Pharmacists and the Infectious Diseases Society of America, for serious MRSA infections with an assumed MIC of 1 mg/L, an AUC/MIC ratio of 400 to 600 should be targeted and trough-only monitoring is no longer recommended [50]. Previously considered to be an adequate surrogate for AUC/MIC, trough concentrations by themselves are unable to fully represent the different possible PK profiles they are a part of. Instead, one of the two following AUC-based dosing and monitoring methods is recommended. The first is to measure a steady-state peak concentration (1–2 h post-infusion) and a trough concentration, ideally within the same interval. These should then be used in first-order PK equations to calculate the AUC. The second, more preferred approach is to collect the previously described samples in a Bayesian software program containing a PK model rich in vancomycin data. A standard regimen of 15 to 20 mg/kg every 8–12 h using actual body weight was recommended for those with normal renal function. However, Elder and colleagues conducted a retrospective study involving patients afflicted with thermal or inhalation injuries [51]. In a subgroup analysis conducted to control for altered renal status, they observed that the average patient with at least 10% of their total body surface area burned needed 64.7 mg/kg/day to achieve a goal trough of 15 to 20 mg/L. Any patient with greater than average requirements already exceeds the standard intermittent dosing regimen, further emphasizing how important monitoring continues to be for this drug. In serious infections, a 20 to 35 mg/kg loading dose can be considered up to a maximum of 3000 mg [50]. Obese patients may also receive doses up to this maximum, though the guidelines note that dosing is not typically more than 4500 mg/day. Continuous infusions up to 60 mg/kg and targeting a steady-state concentration of 20 to 25 mg/L were suggested. In conjunction with the new monitoring recommendations, vancomycin dosing will hopefully now be able to better contend with the enhanced drug clearance seen in ARC patients.

Another option is to switch antibiotics. For instance, a suitable substitution for vancomycin would be linezolid. Linezolid is primarily eliminated through a nonrenal pathway and is instead oxidized [52]. Depending on the publication, linezolid either performs the same or better than vancomycin in treating MRSA pneumonia and skin and soft tissue infections [53,54,55]. The major defining adverse effect of this drug is the reversible thrombocytopenia associated with two or more weeks of therapy [56]. More concerning, a recent 10-year retrospective study conducted in ICU patients concluded that those with linezolid-induced thrombocytopenia also had increased mortality [57]. However, renal impairment and renal replacement therapy appear to be risk factors for this drug-related thrombocytopenia [58,59], suggesting that this concern may not be applicable to patients with ARC. Of note, a recent study observed that linezolid clearance was still exceptionally elevated in those with ARC and the investigators concluded that a continuous confusion at 75 mg/h would be necessary to remain at concentrations of 2 mg/L or above [60]. Thus, even for drugs with non-renal elimination pathways, dosing adjustments may still need to be made. 

### 4.3. Additional Agents

Aminoglycosides are concentration-dependent agents and require a C_max_/MIC of 10 [61]. Two population pharmacokinetic studies focusing on amikacin concluded that ARC patients likely require increased dosing regimens [62,63]. Another pharmacokinetic modeling study in pediatric ICU patients who were administered tobramycin or gentamicin observed significantly lower median 24-h AUC (45.27 vs. 56.95 mg*h/L, *p* < 0.01) [64]. Colistin, known for its nephrotoxicity, has been studied retrospectively in ICU patients afflicted by multidrug-resistant Gram-negative infections [65]. Aitullina and colleagues observed that patients with ARC usually received the standard daily dose of 9 million units. Their median cumulative doses, however, trended higher and they received therapy for a longer duration relative to non-ARC patients, though not to a statistically significant degree. Although ARC might theoretically provide a renal protective factor, this particular study did not produce results to support this. Finally, there is little data regarding the use of antifungals in patients with ARC and guidance is based on previously established practices and PK/PD target attainment under non-ARC conditions. For instance, because fluconazole undergoes significant renal elimination, increasing the dose has been recommended, but to what degree has not been specified [66,67]. Therapeutic drug monitoring for mold-active triazoles and flucytosine appears to be the main recommendation, though it should be noted that doing so is the standard for these medications [67,68].

Newer agents also have potential. Approved in 2019, cefiderocol provides dosing adjustment guidance for patients with elevated creatinine clearances of 120 mL/min or higher, increasing the standard approved dosage of 2 g every 8 h to 2 g every 6 h [69]. Including dosing information for those with enhanced renal function was a first and may potentially encourage drugs currently in the pipeline to follow suit. Interim safety results from a Phase 1 PK study reported that twice the standard dosing of ceftolozane/tazobactam, or 3 g every 8 h, was safe and generated sufficient exposure in twelve critically ill pneumonia patients with a mean creatinine clearance of 134.6 mL/min [70]. The exposures from this increased regimen were confirmed to be adequate upon completion of this study [71]. A population pharmacokinetic analysis that took ARC into consideration was performed for imipenem/relebactam [72]. It found a more than 90% probability of target attainment (PTA) when using a 500/250 mg dose against an imipenem MIC of 4 mg/L when creatinine clearance is 90 to 250 mL/min. Ceftazidime/avibactam achieved a greater than 90% PTA for using 2000/500 mg every 8 h in patients with a creatinine clearance over 80 mL/min against an MIC of 8 mg/L [73]. Augmented renal clearance defined at 130 mL/min was also considered in a daptomycin population pharmacokinetic study [74]. These recently conducted studies provide additional insights regarding drug exposure optimization in the setting of ARC. Moreover, the inclusion of ARC dosing guidance in the initial version of the cefiderocol package insert at the time of drug approval paves the way for the optimal use of this compound as well as sets a new standard for consideration of this important target population.

## 5. Conclusions

Augmented renal clearance is a common condition for ICU patients. It can eliminate antibiotics so effectively that subtherapeutic concentrations and clinical failure become real possibilities. As such, it is important to accurately and regularly assess kidney function in these patients, especially with 8-h urine collection studies in those that are already catheterized so that the healthcare team can respond appropriately. Given the frequent need for antibiotics and the likely chance their concentrations will be impacted, dosing regimens should be optimized, either by maximizing the dose or using prolonged infusions, or making the decision to switch to another agent.

## Figures and Tables

**Table 1:** Equations used to estimate renal function ^a^.

1. Cockcroft–Gault (CG) [2]: $\mathrm{CrCl}=\frac{\left[140-age\;\left(years\right)\right]\times weight\;\left(kg\right)}{72\times SCr\;\left(mg/dL\right)}$ × 0.85 (if female) 2. Modification of Diet in Renal Disease (MDRD) [3]: eGFR = 175 × S_Cr_^−^^1.154^ × age^−0.203^ × 0.742 (if female) × 1.212 (if black) 3. Chronic Kidney Disease Epidemiology Collaboration (CKD-EPI) [4]: eGFR = 141 × min (S_Cr_/κ, 1)^α^ × max (S_Cr_/κ, 1)^−1.209^ × 0.993^age^ × 1.018 (if female) × 1.159 (if black)

^a^ CrCl, creatinine clearance; S_Cr_, serum creatinine; eGFR, estimated glomerular filtration rate; κ, 0.7 (if female) or 0.9 (if male); α, −0.329 (if female) or −0.411 (if male); min, minimum between S_Cr_/κ and 1; max, maximum between S_Cr_/κ and 1.

**Table 2:** Strategies for antibiotic use in patients with augmented renal clearance.

1. Use maximum approved dosing regimen
2. Administer doses in a prolonged or continuous infusion
3. Therapeutic drug monitoring
4. Switch to an alternative agent that is not largely renally eliminated
